# 12-{[4-(4-Bromo­phen­yl)piperazin-1-yl]meth­yl}-9α-hy­droxy-4,8-dimethyl-3,14-dioxatri­cyclo­[9.3.0.0^2,4^]tetra­dec-7-en-13-one

**DOI:** 10.1107/S1600536814006473

**Published:** 2014-03-29

**Authors:** Mohamed Loubidi, Ahmed Benharref, Lahcen El Ammari, Mohamed Saadi, Moha Berraho

**Affiliations:** a Laboratoire de Chimie Biomoleculaire, Substances Naturelles et Réactivité, URAC16, Faculté des Sciences Semlalia, BP 2390 Bd My Abdellah, 40000 Marrakech, Morocco; b Laboratoire de Chimie du Solide Appliquée, Faculté des Sciences, Université Mohammed V-Agdal, BP 1014, Avenue Ibn Battouta, Rabat, Morocco

## Abstract

The title compound, C_25_H_33_BrN_2_O_4_, was synthesized from 9α-hy­droxy­parthenolide (9α-hy­droxy-4,8-dimethyl-12-methylen-3,14-dioxa-tri­cyclo­[9.3.0.0^2,4^]tetra­dec-7-en-13-one), which was isolated from the chloro­form extract of the aerial parts of *Anvillea radiata*. The mol­ecule is built up from two fused five- and ten-membered rings with an additional ep­oxy ring system and a bromo­phenyl­piperazine group as a substituent. The ten-membered ring adopts an approximate chair–chair–chair conformation, while the piperazine ring displays a chair conformation and the five-membered ring shows an envelope conformation with the C atom closest to the hy­droxy group forming the flap. An intra­molecular O—H⋯N hydrogen bond stabilizes the mol­ecular conformation. The crystal packing features C—H⋯O hydrogen bonds, which link the mol­ecules into zigzag chains running along the *b-*axis direction.

## Related literature   

For background to the medicinal uses of the plant *Anvillea radiata*, see: Abdel Sattar *et al.* (1996 ▶); El Hassany *et al.* (2004 ▶); Qureshi *et al.* (1990 ▶). For the reactivity of this sesquiterpene, see: Neukirch *et al.* (2003 ▶); Hwang *et al.* (2006 ▶); Neelakantan *et al.* (2009 ▶). For the synthetic procedure, see: Moumou *et al.* (2010 ▶). For conformational analysis, see: Cremer & Pople (1975 ▶)
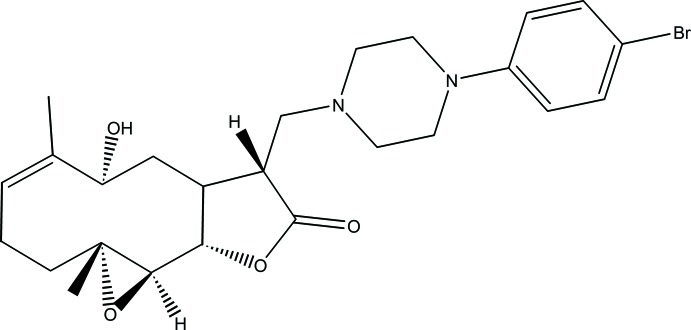


## Experimental   

### 

#### Crystal data  

C_25_H_33_BrN_2_O_4_
*M* *_r_* = 505.44
Monoclinic, 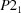

*a* = 9.6790 (4) Å
*b* = 7.0710 (3) Å
*c* = 17.3117 (7) Å
β = 94.872 (2)°
*V* = 1180.54 (8) Å^3^
*Z* = 2
Mo *K*α radiation
μ = 1.78 mm^−1^
*T* = 296 K
0.5 × 0.03 × 0.03 mm

#### Data collection  

Bruker X8 APEX diffractometer
Absorption correction: multi-scan (*SADABS*; Bruker, 2009 ▶) *T*_min_ = 0.634, *T*_max_ = 0.746
14527 measured reflections
5899 independent reflections
5216 reflections with *I* > 2σ(*I*)
*R*_int_ = 0.024

#### Refinement  

*R*[*F* ^2^ > 2σ(*F* ^2^)] = 0.030
*wR*(*F* ^2^) = 0.076
*S* = 1.03
5899 reflections
292 parameters
1 restraint
H-atom parameters constrained
Δρ_max_ = 0.44 e Å^−3^
Δρ_min_ = −0.44 e Å^−3^
Absolute structure: Flack & Bernardinelli (2000 ▶), 2614 Friedel pairs
Absolute structure parameter: 0.007 (5)

### 

Data collection: *APEX2* (Bruker, 2009 ▶); cell refinement: *SAINT* (Bruker, 2009 ▶); data reduction: *SAINT*; program(s) used to solve structure: *SHELXS97* (Sheldrick, 2008 ▶); program(s) used to refine structure: *SHELXL97* (Sheldrick, 2008 ▶); molecular graphics: *ORTEP-3 for Windows* (Farrugia, 2012 ▶); software used to prepare material for publication: *PLATON* (Spek, 2009 ▶) and *publCIF* (Westrip, 2010 ▶).

## Supplementary Material

Crystal structure: contains datablock(s) I, global. DOI: 10.1107/S1600536814006473/bt6970sup1.cif

Structure factors: contains datablock(s) I. DOI: 10.1107/S1600536814006473/bt6970Isup2.hkl

Supporting information file. DOI: 10.1107/S1600536814006473/bt6970Isup3.cml

CCDC reference: 993318

Additional supporting information:  crystallographic information; 3D view; checkCIF report

## Figures and Tables

**Table 1:** Hydrogen-bond geometry (Å, °)

*D*—H⋯*A*	*D*—H	H⋯*A*	*D*⋯*A*	*D*—H⋯*A*
O4—H4⋯N1	0.82	2.18	2.995 (4)	171
C1—H1⋯O1^i^	0.98	2.52	3.389 (3)	148
C15—H15*C*⋯O1^i^	0.96	2.47	3.410 (3)	167

Symmetry code: (i) 
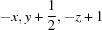
.

**Fig. 1.**
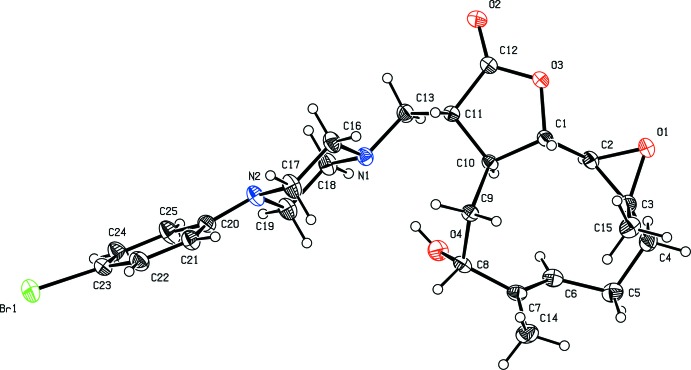
: Molecular structure of the title compound with the atom-labelling scheme. Displacement ellipsoids are drawn at the 30% probability level. H atoms are represented as small spheres of arbitrary radii.

**Fig. 2.**
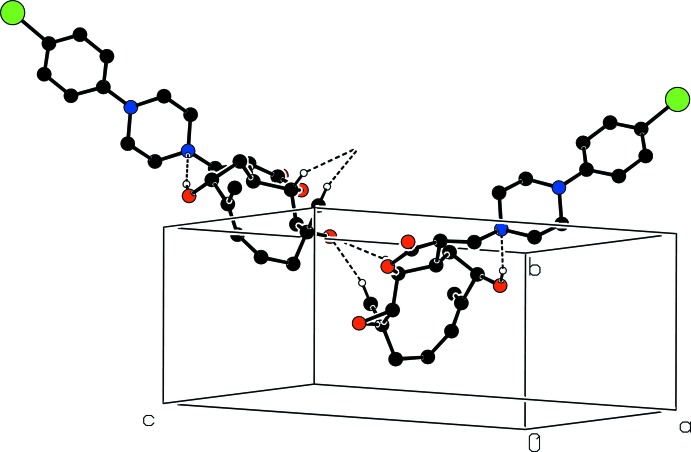
: Packing view showing the C–H···O and O–H···N hydrogen bonds as dashed lines. H atoms not involved in hydrogen bonding have been omitted for clarity.

## supplementary crystallographic information

### 1. Comment

The natural sesquiterpene lactone 9α-hydroxypartenolide is the main constituent of the chloroform extract of the aerial parts of *Anvillea radiata* (El Hassany *et al.*, 2004) and of *Anvillea garcini* (Abdel Sattar *et al.*, 1996). The reactivity of this sesquiterpene lactone and its derivatives has been the subject of several studies (Hwang *et al.*, 2006; Neelakantan *et al.*, 2009; Moumou *et al.*, 2010), in order to prepare products with a high added value that can be used in the pharmacological industry. In this context, we have treated 9α-hydroxyparthenolide with an equivalent amount of 1- (4-bromophenyl)piperazine gives the title compound. The molecule contains a fused ring system and the bromophenyl-pyperazine group as a substituent to the lactone ring. The molecular structure (Fig. 1) shows the lactone ring to adopt an envelope conformation, as indicated by Cremer & Pople (1975) puckering parameters QT = 0.2525 (19) Å and φ2 = 259.3 (4)°. The ten-membered ring displays an approximate chair-chair-chair conformation, while the pyperazine ring has a perfect chair conformation with QT = 0.586 (2) Å, θ = 177.96 (18) and φ2 = 165 (4)°. In the crystal, C—H···O hydrogen bonding links the molecules to zigzag chains running along the b-axis (Table 1, Fig. 2). In addition, an intramolecular O4—H4···N1 hydrogen bond is also observed. Owing to the presence of a Br atom, the absolute configuration could be fully confirmed, by refining the Flack parameter (Flack & Bernardinelli, 2000) as C1(S), C2(R) C3(R), C8(R), C10(S) and C11(R).

### 2. Experimental

The mixture of 9?-hydoxypartenolide (9α-hydroxy-4,8-dimethyl- 12-methylene- 3,14-dioxatricyclo[9.3.0.0^2^,^4^]tetradec-7-en-13-one) (1 g, 3.8 mmol) and one equivalent of 1-(4-bromophenyl-piperazine) in EtOH (20 ml) was stirred for twelve hours at room temperature. Then, the reaction was stopped by adding water (10 ml) and the solution was extracted with chloroform (3 *x* 20 ml). The combined organic layers were dried over anhydrous MgSO_4_, filtered and concentrated under vacuum to give 1.5 g (3 mmol) of the title compound (yield: 79%). Recrystallization was performed from in ethyl acetate.

### 3. Refinement

All H atoms were fixed geometrically and treated as riding with C—H = 0.96 Å (methyl), 0.97 Å (methylene), 0.98 Å (methine) and O–H = 0.82 Å with *U*_iso_(H) = 1.2*U*_eq_ (methylene, methine) or *U*_iso_(H) = 1.5*U*_eq_(C_methyl_, O). The torsion angles about the C-C_methyl_ and C-O bonds were refined.

### Crystal data

**Table:**

C_25_H_33_BrN_2_O_4_	*F*(000) = 528
*M**_r_* = 505.44	*D*_x_ = 1.422 Mg m^−^^3^
Monoclinic, *P*2_1_	Mo *K*α radiation, λ = 0.71073 Å
Hall symbol: P 2yb	Cell parameters from 5899 reflections
*a* = 9.6790 (4) Å	θ = 2.4–28.7°
*b* = 7.0710 (3) Å	µ = 1.78 mm^−^^1^
*c* = 17.3117 (7) Å	*T* = 296 K
β = 94.872 (2)°	Box, colourless
*V* = 1180.54 (8) Å^3^	0.5 × 0.03 × 0.03 mm
*Z* = 2	

### Data collection

**Table:**

Bruker X8 APEX diffractometer	5899 independent reflections
Radiation source: fine-focus sealed tube	5216 reflections with *I* > 2σ(*I*)
Graphite monochromator	*R*_int_ = 0.024
φ and ω scans	θ_max_ = 28.7°, θ_min_ = 2.4°
Absorption correction: multi-scan (*SADABS*; Bruker, 2009)	*h* = −12→13
*T*_min_ = 0.634, *T*_max_ = 0.746	*k* = −9→9
14527 measured reflections	*l* = −23→23

### Refinement

**Table:**

Refinement on *F*^2^	Secondary atom site location: difference Fourier map
Least-squares matrix: full	Hydrogen site location: inferred from neighbouring sites
*R*[*F*^2^ > 2σ(*F*^2^)] = 0.030	H-atom parameters constrained
*wR*(*F*^2^) = 0.076	*w* = 1/[σ^2^(*F*_o_^2^) + (0.0313*P*)^2^] where *P* = (*F*_o_^2^ + 2*F*_c_^2^)/3
*S* = 1.03	(Δ/σ)_max_ = 0.001
5899 reflections	Δρ_max_ = 0.44 e Å^−^^3^
292 parameters	Δρ_min_ = −0.44 e Å^−^^3^
1 restraint	Absolute structure: Flack & Bernardinelli (2000), 2614 Friedel pairs
Primary atom site location: structure-invariant direct methods	Absolute structure parameter: 0.007 (5)

### Special details

**Table:**

Geometry. All s.u.'s (except the s.u. in the dihedral angle between two l.s. planes) are estimated using the full covariance matrix. The cell s.u.'s are taken into account individually in the estimation of s.u.'s in distances, angles and torsion angles; correlations between s.u.'s in cell parameters are only used when they are defined by crystal symmetry. An approximate (isotropic) treatment of cell s.u.'s is used for estimating s.u.'s involving l.s. planes.

### Fractional atomic coordinates and isotropic or equivalent isotropic displacement parameters (Å^2^)

**Table:**

	*x*	*y*	*z*	*U*_iso_*/*U*_eq_	
Br1	0.81297 (2)	0.79383 (4)	−0.080172 (12)	0.06016 (9)	
O1	0.06025 (14)	−0.47571 (19)	0.48432 (7)	0.0400 (3)	
O3	0.29861 (12)	−0.1832 (2)	0.50486 (6)	0.0367 (3)	
O4	0.34442 (13)	−0.25359 (19)	0.21350 (8)	0.0418 (3)	
H4	0.4035	−0.1779	0.2306	0.063*	
N1	0.53704 (14)	0.0338 (2)	0.29071 (8)	0.0302 (3)	
N2	0.63915 (15)	0.2755 (2)	0.17458 (8)	0.0351 (3)	
C1	0.20134 (15)	−0.2018 (3)	0.43565 (8)	0.0280 (3)	
H1	0.1227	−0.1152	0.4376	0.034*	
O2	0.51010 (17)	−0.0713 (3)	0.53716 (9)	0.0637 (5)	
C11	0.40639 (17)	−0.0352 (2)	0.40481 (10)	0.0301 (3)	
H11	0.3778	0.0978	0.4024	0.036*	
C10	0.28736 (16)	−0.1544 (2)	0.36682 (9)	0.0257 (3)	
H10	0.3270	−0.2728	0.3492	0.031*	
C2	0.15415 (18)	−0.4034 (2)	0.43110 (10)	0.0302 (3)	
H2	0.2277	−0.4925	0.4205	0.036*	
C9	0.20719 (17)	−0.0612 (2)	0.29633 (10)	0.0275 (3)	
H9A	0.2462	0.0630	0.2885	0.033*	
H9B	0.1115	−0.0439	0.3075	0.033*	
C20	0.66722 (19)	0.3843 (3)	0.11021 (10)	0.0333 (4)	
C15	−0.1053 (2)	−0.3330 (3)	0.38362 (13)	0.0416 (4)	
H15A	−0.1899	−0.3920	0.3961	0.062*	
H15B	−0.1117	−0.3022	0.3294	0.062*	
H15C	−0.0903	−0.2196	0.4136	0.062*	
C8	0.21012 (17)	−0.1764 (2)	0.22029 (9)	0.0311 (3)	
H8	0.1889	−0.0899	0.1766	0.037*	
C7	0.09765 (18)	−0.3259 (3)	0.21770 (10)	0.0315 (3)	
C14	−0.0380 (2)	−0.2547 (3)	0.18031 (13)	0.0460 (5)	
H14A	−0.1099	−0.3441	0.1886	0.069*	
H14B	−0.0312	−0.2393	0.1257	0.069*	
H14C	−0.0595	−0.1353	0.2027	0.069*	
C3	0.01452 (19)	−0.4672 (2)	0.40199 (10)	0.0319 (4)	
C16	0.5343 (2)	0.2401 (3)	0.29620 (11)	0.0392 (4)	
H16A	0.6185	0.2834	0.3253	0.047*	
H16B	0.4563	0.2783	0.3242	0.047*	
C17	0.5224 (2)	0.3318 (3)	0.21754 (11)	0.0421 (5)	
H17A	0.4361	0.2940	0.1891	0.051*	
H17B	0.5215	0.4683	0.2233	0.051*	
C5	0.0058 (2)	−0.6297 (2)	0.27166 (12)	0.0395 (4)	
H5A	0.0218	−0.7510	0.2478	0.047*	
H5B	−0.0839	−0.5833	0.2507	0.047*	
C6	0.1169 (2)	−0.4925 (2)	0.25258 (10)	0.0343 (4)	
H6	0.2081	−0.5278	0.2666	0.041*	
C13	0.54475 (19)	−0.0520 (3)	0.36882 (11)	0.0364 (4)	
H13A	0.6166	0.0107	0.4020	0.044*	
H13B	0.5696	−0.1844	0.3653	0.044*	
C21	0.5923 (2)	0.5468 (3)	0.08862 (11)	0.0379 (4)	
H21	0.5133	0.5762	0.1133	0.046*	
C22	0.6333 (2)	0.6657 (3)	0.03105 (11)	0.0420 (4)	
H22	0.5826	0.7745	0.0179	0.050*	
C18	0.6578 (2)	−0.0185 (3)	0.24953 (12)	0.0387 (4)	
H18A	0.6627	−0.1551	0.2451	0.046*	
H18B	0.7419	0.0249	0.2786	0.046*	
C4	0.0055 (2)	−0.6550 (2)	0.36027 (12)	0.0398 (4)	
H4A	−0.0788	−0.7194	0.3717	0.048*	
H4B	0.0835	−0.7333	0.3790	0.048*	
C12	0.4169 (2)	−0.0961 (3)	0.48858 (11)	0.0389 (4)	
C24	0.8214 (2)	0.4589 (4)	0.01092 (13)	0.0514 (5)	
H24	0.8976	0.4280	−0.0158	0.062*	
C23	0.7487 (2)	0.6225 (3)	−0.00648 (11)	0.0414 (4)	
C19	0.6466 (2)	0.0693 (3)	0.16953 (11)	0.0383 (4)	
H19A	0.7265	0.0336	0.1427	0.046*	
H19B	0.5642	0.0220	0.1399	0.046*	
C25	0.7808 (2)	0.3408 (3)	0.06823 (12)	0.0471 (5)	
H25	0.8299	0.2297	0.0793	0.057*	

### Atomic displacement parameters (Å^2^)

**Table:**

	*U*^11^	*U*^22^	*U*^33^	*U*^12^	*U*^13^	*U*^23^
Br1	0.06273 (15)	0.07268 (15)	0.04532 (12)	−0.01813 (12)	0.00605 (9)	0.02022 (11)
O1	0.0448 (8)	0.0446 (7)	0.0312 (7)	−0.0100 (6)	0.0071 (6)	0.0077 (5)
O3	0.0389 (6)	0.0481 (7)	0.0232 (5)	−0.0105 (6)	0.0036 (4)	0.0038 (5)
O4	0.0341 (7)	0.0539 (8)	0.0390 (7)	0.0043 (6)	0.0129 (6)	−0.0079 (6)
N1	0.0268 (7)	0.0342 (7)	0.0301 (7)	0.0026 (6)	0.0059 (6)	0.0041 (6)
N2	0.0405 (8)	0.0358 (7)	0.0306 (7)	0.0071 (7)	0.0129 (6)	0.0049 (6)
C1	0.0287 (7)	0.0316 (7)	0.0241 (7)	−0.0003 (7)	0.0043 (5)	0.0015 (7)
O2	0.0577 (10)	0.0973 (13)	0.0334 (8)	−0.0328 (10)	−0.0125 (7)	0.0183 (9)
C11	0.0295 (9)	0.0359 (8)	0.0245 (8)	−0.0029 (7)	0.0003 (6)	0.0029 (6)
C10	0.0262 (8)	0.0286 (7)	0.0227 (7)	0.0038 (6)	0.0043 (6)	0.0011 (5)
C2	0.0328 (9)	0.0299 (7)	0.0283 (8)	0.0024 (7)	0.0045 (7)	0.0058 (6)
C9	0.0280 (8)	0.0271 (7)	0.0274 (8)	0.0050 (6)	0.0014 (6)	0.0022 (6)
C20	0.0325 (9)	0.0395 (9)	0.0281 (9)	0.0016 (7)	0.0033 (7)	0.0017 (7)
C15	0.0328 (10)	0.0442 (10)	0.0484 (12)	0.0042 (8)	0.0058 (8)	−0.0078 (9)
C8	0.0338 (8)	0.0362 (9)	0.0232 (7)	0.0060 (7)	0.0028 (6)	0.0036 (7)
C7	0.0332 (9)	0.0361 (8)	0.0252 (8)	0.0059 (7)	0.0015 (7)	−0.0057 (6)
C14	0.0394 (10)	0.0482 (11)	0.0478 (11)	0.0022 (8)	−0.0109 (8)	0.0045 (9)
C3	0.0338 (9)	0.0315 (8)	0.0310 (9)	−0.0022 (7)	0.0065 (7)	0.0005 (7)
C16	0.0476 (11)	0.0376 (9)	0.0346 (9)	0.0001 (7)	0.0158 (8)	−0.0007 (7)
C17	0.0503 (11)	0.0362 (10)	0.0428 (10)	0.0128 (8)	0.0219 (9)	0.0053 (8)
C5	0.0468 (11)	0.0280 (8)	0.0428 (11)	0.0010 (7)	−0.0005 (8)	−0.0059 (7)
C6	0.0372 (9)	0.0337 (8)	0.0321 (9)	0.0074 (7)	0.0035 (7)	−0.0058 (7)
C13	0.0274 (9)	0.0472 (10)	0.0343 (9)	−0.0015 (7)	0.0016 (7)	0.0097 (8)
C21	0.0371 (10)	0.0421 (9)	0.0353 (9)	0.0033 (8)	0.0073 (8)	0.0044 (8)
C22	0.0491 (12)	0.0412 (9)	0.0353 (10)	0.0011 (9)	0.0019 (8)	0.0077 (8)
C18	0.0330 (9)	0.0410 (10)	0.0435 (11)	0.0087 (8)	0.0119 (8)	0.0073 (8)
C4	0.0444 (11)	0.0303 (9)	0.0447 (11)	−0.0045 (7)	0.0047 (8)	0.0019 (7)
C12	0.0408 (11)	0.0483 (10)	0.0273 (9)	−0.0103 (8)	0.0003 (7)	0.0050 (8)
C24	0.0478 (12)	0.0690 (14)	0.0397 (11)	0.0096 (11)	0.0177 (9)	0.0107 (10)
C23	0.0443 (11)	0.0519 (12)	0.0275 (9)	−0.0110 (9)	−0.0005 (8)	0.0069 (8)
C19	0.0432 (11)	0.0365 (9)	0.0374 (10)	0.0094 (8)	0.0161 (8)	0.0013 (7)
C25	0.0480 (11)	0.0559 (13)	0.0396 (10)	0.0154 (9)	0.0160 (9)	0.0120 (9)

### Geometric parameters (Å, º)

**Table:**

Br1—C23	1.9020 (19)	C8—H8	0.9800
O1—C2	1.442 (2)	C7—C6	1.329 (3)
O1—C3	1.457 (2)	C7—C14	1.500 (3)
O3—C12	1.351 (2)	C14—H14A	0.9600
O3—C1	1.465 (2)	C14—H14B	0.9600
O4—C8	1.424 (2)	C14—H14C	0.9600
O4—H4	0.8200	C3—C4	1.510 (3)
N1—C16	1.462 (3)	C16—C17	1.504 (3)
N1—C18	1.467 (2)	C16—H16A	0.9700
N1—C13	1.478 (2)	C16—H16B	0.9700
N2—C20	1.400 (2)	C17—H17A	0.9700
N2—C17	1.460 (2)	C17—H17B	0.9700
N2—C19	1.462 (3)	C5—C6	1.505 (3)
C1—C2	1.497 (3)	C5—C4	1.545 (3)
C1—C10	1.5473 (19)	C5—H5A	0.9700
C1—H1	0.9800	C5—H5B	0.9700
O2—C12	1.193 (3)	C6—H6	0.9300
C11—C12	1.508 (2)	C13—H13A	0.9700
C11—C13	1.529 (2)	C13—H13B	0.9700
C11—C10	1.530 (2)	C21—C22	1.387 (3)
C11—H11	0.9800	C21—H21	0.9300
C10—C9	1.538 (2)	C22—C23	1.374 (3)
C10—H10	0.9800	C22—H22	0.9300
C2—C3	1.473 (3)	C18—C19	1.513 (3)
C2—H2	0.9800	C18—H18A	0.9700
C9—C8	1.550 (2)	C18—H18B	0.9700
C9—H9A	0.9700	C4—H4A	0.9700
C9—H9B	0.9700	C4—H4B	0.9700
C20—C21	1.393 (3)	C24—C23	1.373 (3)
C20—C25	1.402 (2)	C24—C25	1.379 (3)
C15—C3	1.511 (3)	C24—H24	0.9300
C15—H15A	0.9600	C19—H19A	0.9700
C15—H15B	0.9600	C19—H19B	0.9700
C15—H15C	0.9600	C25—H25	0.9300
C8—C7	1.516 (3)		
			
C2—O1—C3	61.05 (11)	O1—C3—C15	113.30 (14)
C12—O3—C1	111.57 (12)	C2—C3—C15	123.01 (16)
C8—O4—H4	109.5	C4—C3—C15	116.06 (17)
C16—N1—C18	107.64 (14)	N1—C16—C17	111.83 (16)
C16—N1—C13	110.47 (15)	N1—C16—H16A	109.2
C18—N1—C13	111.14 (14)	C17—C16—H16A	109.2
C20—N2—C17	117.81 (15)	N1—C16—H16B	109.2
C20—N2—C19	119.11 (14)	C17—C16—H16B	109.2
C17—N2—C19	110.26 (14)	H16A—C16—H16B	107.9
O3—C1—C2	107.50 (14)	N2—C17—C16	109.89 (15)
O3—C1—C10	105.06 (12)	N2—C17—H17A	109.7
C2—C1—C10	110.33 (13)	C16—C17—H17A	109.7
O3—C1—H1	111.2	N2—C17—H17B	109.7
C2—C1—H1	111.2	C16—C17—H17B	109.7
C10—C1—H1	111.2	H17A—C17—H17B	108.2
C12—C11—C13	112.33 (15)	C6—C5—C4	110.75 (16)
C12—C11—C10	104.05 (13)	C6—C5—H5A	109.5
C13—C11—C10	115.82 (14)	C4—C5—H5A	109.5
C12—C11—H11	108.1	C6—C5—H5B	109.5
C13—C11—H11	108.1	C4—C5—H5B	109.5
C10—C11—H11	108.1	H5A—C5—H5B	108.1
C11—C10—C9	114.56 (13)	C7—C6—C5	126.55 (18)
C11—C10—C1	102.89 (13)	C7—C6—H6	116.7
C9—C10—C1	115.83 (13)	C5—C6—H6	116.7
C11—C10—H10	107.7	N1—C13—C11	111.29 (14)
C9—C10—H10	107.7	N1—C13—H13A	109.4
C1—C10—H10	107.7	C11—C13—H13A	109.4
O1—C2—C3	59.99 (11)	N1—C13—H13B	109.4
O1—C2—C1	120.69 (14)	C11—C13—H13B	109.4
C3—C2—C1	125.25 (15)	H13A—C13—H13B	108.0
O1—C2—H2	113.5	C22—C21—C20	121.23 (17)
C3—C2—H2	113.5	C22—C21—H21	119.4
C1—C2—H2	113.5	C20—C21—H21	119.4
C10—C9—C8	113.85 (13)	C23—C22—C21	119.91 (19)
C10—C9—H9A	108.8	C23—C22—H22	120.0
C8—C9—H9A	108.8	C21—C22—H22	120.0
C10—C9—H9B	108.8	N1—C18—C19	110.06 (15)
C8—C9—H9B	108.8	N1—C18—H18A	109.6
H9A—C9—H9B	107.7	C19—C18—H18A	109.6
C21—C20—N2	122.36 (15)	N1—C18—H18B	109.6
C21—C20—C25	117.07 (17)	C19—C18—H18B	109.6
N2—C20—C25	120.38 (16)	H18A—C18—H18B	108.2
C3—C15—H15A	109.5	C3—C4—C5	111.61 (15)
C3—C15—H15B	109.5	C3—C4—H4A	109.3
H15A—C15—H15B	109.5	C5—C4—H4A	109.3
C3—C15—H15C	109.5	C3—C4—H4B	109.3
H15A—C15—H15C	109.5	C5—C4—H4B	109.3
H15B—C15—H15C	109.5	H4A—C4—H4B	108.0
O4—C8—C7	112.85 (15)	O2—C12—O3	121.57 (17)
O4—C8—C9	110.74 (14)	O2—C12—C11	128.41 (17)
C7—C8—C9	109.07 (12)	O3—C12—C11	109.99 (16)
O4—C8—H8	108.0	C23—C24—C25	119.72 (18)
C7—C8—H8	108.0	C23—C24—H24	120.1
C9—C8—H8	108.0	C25—C24—H24	120.1
C6—C7—C14	125.23 (18)	C24—C23—C22	120.37 (18)
C6—C7—C8	122.16 (17)	C24—C23—Br1	119.54 (15)
C14—C7—C8	112.24 (16)	C22—C23—Br1	120.05 (16)
C7—C14—H14A	109.5	N2—C19—C18	110.77 (16)
C7—C14—H14B	109.5	N2—C19—H19A	109.5
H14A—C14—H14B	109.5	C18—C19—H19A	109.5
C7—C14—H14C	109.5	N2—C19—H19B	109.5
H14A—C14—H14C	109.5	C18—C19—H19B	109.5
H14B—C14—H14C	109.5	H19A—C19—H19B	108.1
O1—C3—C2	58.95 (11)	C24—C25—C20	121.58 (19)
O1—C3—C4	115.59 (15)	C24—C25—H25	119.2
C2—C3—C4	116.57 (15)	C20—C25—H25	119.2

### Hydrogen-bond geometry (Å, º)

**Table:**

*D*—H···*A*	*D*—H	H···*A*	*D*···*A*	*D*—H···*A*
O4—H4···N1	0.82	2.18	2.995 (4)	171
C1—H1···O1^i^	0.98	2.52	3.389 (3)	148
C15—H15*C*···O1^i^	0.96	2.47	3.410 (3)	167

Symmetry code: (i) −*x*, *y*+1/2, −*z*+1.
